# Coping strategies among medical and paramedic frontline healthcare workers during the coronavirus pandemic

**DOI:** 10.1192/j.eurpsy.2022.1301

**Published:** 2022-09-01

**Authors:** I. Kammoun, O. Maatouk, R. Kammoun, M. Shiri, H. Nefzi, K. Ben Salah, M. Karoui, F. Ellouz

**Affiliations:** Razi hospital, Psychiatry G Department, Mannouba, Tunisia

**Keywords:** covid 19, coping strategies, healthcare workers, Anxiety and depression

## Abstract

**Introduction:**

The epidemic of COVID-19 has affected the psychological health of people, especially frontline medical and paramedical staff. Several coping strategies have been used to combat the impact of this virus on their lives.

**Objectives:**

Describe the impact of coronavirus on mental health and identify coping strategies

**Methods:**

We carried out a cross-sectional, descriptive and analytical study, conducted over a period of two months ( september and october 2020), in 22 hospitals in Tunisia, including frontline medical and paramedical staff. To evaluate anxiety and depression, we used the Beck Inventory. To identify coping strategies, we used the Brief COPE.

**Results:**

We collected 78 professionals. The mean age was 29.86+-5.4. The majority were medical residents (67.9%) working in covid units in 39.7% of cases. The rythm of work was daily in almost half of the cases, giving direct care to the patients tested positive in 76.9%. More than half had not received adequate training, and protective equipment was available in only 50% of cases. We found 35.9% of the staff who had to move for fear of infecting their families. More than half of the frontline staff were victims of stigma (57.7%). Depression and anxiety were tested minor in 40%. The most used coping strategy in the face of this distressing virus was social support (64.1%) followed by emotion-focused mechanisms (53,8%). Social support strategy was significantly correlated with prevention of anxiety (p=0.048)

**Conclusions:**

Participants practiced and recommended various coping strategies to deal with stress, depression and anxiety emerging from COVID-19 pandemic.

**Disclosure:**

No significant relationships.

